# Fracture Non-Union in Osteoporotic Bones: Current Practice and Future Directions

**DOI:** 10.7759/cureus.69778

**Published:** 2024-09-20

**Authors:** Chijioke Orji, Charles Ojo, Daniel E Onobun, Kenechukwu Igbokwe, Farihah Khaliq, Reginald Ononye

**Affiliations:** 1 Department of Trauma and Orthopedics, Liverpool University Hospitals NHS Foundation Trust, Liverpool, GBR; 2 Department of Emergency Medicine, United Lincolnshire Hospitals NHS Trust, Boston, GBR; 3 Department of Neurological Surgery, Wellington Neurosurgery Centre, Abuja, NGA; 4 Department of Neurology, Wellington Hospital, Abuja, NGA; 5 Department of Trauma and Orthopedics, Aintree University Hospital, Liverpool, GBR

**Keywords:** geriatrics, nonunion fracture, osteoporosis, teriparatide, vitamin d

## Abstract

Given the compromised bone quality and altered healing environment, fracture non-union in osteoporotic bones presents a complex challenge in orthopedics. As global populations age, the incidence of osteoporotic fractures rises, leading to increased delayed healing and non-union cases. The pathophysiology underlying non-union in osteoporotic patients involves impaired bone regeneration, reduced osteoblast function, and poor vascularity. Traditional management strategies - ranging from pharmacological interventions like bisphosphonates and teriparatides to surgical approaches such as bone grafting and mechanical fixation - often yield limited success due to the weakened bone structure. Recent advances, however, have introduced novel therapies such as growth factors, stem cell applications, gene therapy, and bioactive scaffolds that offer more targeted and biologically driven solutions. Emerging technologies like three-dimensional printing and nanotechnology further contribute to customized treatment strategies that hold promise for improved outcomes. Diagnostic approaches have also evolved, integrating radiological assessments and biomarkers to identify patients at risk for non-union better. Despite these advancements, challenges remain, including the high costs, technical complexities, and the need for more robust clinical evidence. Future directions involve optimizing these innovative treatments, validating their effectiveness in more extensive clinical trials, and integrating personalized medicine approaches to cater to the individual needs of osteoporotic patients. Overall, integrating these emerging therapeutic strategies alongside traditional practices represents a significant shift towards more effective and personalized management of fracture non-union in osteoporotic bones.

## Introduction and background

Fracture non-union in osteoporotic bones is a critical issue in orthopedics, particularly as global populations age and osteoporosis becomes more prevalent, with a global prevalence of 19.7% [1]. Osteoporotic fractures are uniquely susceptible to non-union due to compromised bone quality, diminished healing capacity, and challenges in achieving stable fixation. The pathophysiology of non-union in these patients involves disrupted bone regeneration, often linked to impaired vascularity, reduced osteoblast function, and mechanical instability [2,3]. While traditional treatments include pharmacological interventions and surgical techniques such as bone grafting and advanced fixation, they cannot address the underlying issues [4]. However, recent advances offer new hope through innovative approaches like biologics, stem cell therapy, gene therapy, and bioactive scaffolds [5-8]. These developments, along with patient-specific strategies and novel technologies like nanomedicine and three-dimensional (3D) printing, pave the way for more effective and personalized management. This article explores the epidemiology, pathophysiology, diagnostic considerations, emerging therapeutic strategies for fracture non-union in osteoporotic bones, and the future directions and clinical implications of these advancements.

## Review

Epidemiology and clinical significance

Fracture non-union in osteoporotic bones represents a significant clinical challenge, particularly given the increasing prevalence of osteoporosis in aging populations. Epidemiologically, the incidence of fracture non-union is notably higher in patients with osteoporosis due to compromised bone quality, leading to impaired healing. Studies estimate that 5-10% of all fractures result in non-union, with osteoporotic fractures particularly prone due to diminished bone density, reduced osteoblastic activity, and poor vascular supply [9]. Clinically, non-union in osteoporotic patients contributes to prolonged disability, increased morbidity, and a higher burden on healthcare resources. The delayed healing process often necessitates more aggressive interventions such as bone grafting, advanced fixation techniques, or bone stimulators, further complicating management [10]. Addressing non-union in osteoporotic bones thus remains critical in improving patient outcomes and reducing long-term complications [11].

Pathophysiology of fracture non-union

The pathophysiology of fracture non-union is multifactorial, involving biological, mechanical, and systemic factors that disrupt the normal bone healing process. Typically, bone healing proceeds through a well-coordinated inflammation, repair, and remodeling sequence. In non-union, however, this sequence is interrupted. A critical factor is the inadequate biological response at the fracture site, often due to impaired vascularity, poor osteogenic activity, or insufficient recruitment of osteoprogenitor cells [2]. Additionally, mechanical instability at the fracture site can lead to excessive micromovement, preventing the formation of a stable callus and promoting fibrous tissue instead of bone [3]. Systemic factors such as malnutrition, smoking, and specific comorbidities like diabetes can further compromise the healing environment by affecting cellular function and the local inflammatory response [11]. As a result, the fracture remains unhealed, leading to the persistence of pain, disability, and the need for more complex interventions.

Diagnostic considerations

Investigations and diagnostic considerations for fracture non-union in osteoporotic bones involve a comprehensive evaluation combining clinical, radiological, and sometimes biochemical assessments (Table 1). Clinically, persistent pain, abnormal mobility at the fracture site, and failure to bear weight after the expected healing period are critical indicators of non-union [12]. Radiologically, plain X-rays remain the initial tool, revealing gaps at the fracture site, sclerosis, or a lack of bridging callus. Advanced imaging techniques such as CT scans provide more detailed views of the fracture morphology. They can better identify signs of non-union, including fibrous tissue or incomplete healing [13]. In cases where the infection is suspected as a contributing factor, MRI or nuclear imaging may be utilized. Additionally, bone turnover markers and metabolic tests are sometimes performed to evaluate the underlying osteoporotic activity and guide treatment strategies [14]. Accurate and timely diagnosis is crucial for formulating an effective management plan, including addressing the mechanical and biological aspects of healing in osteoporotic patients.

**Table 1:** Key diagnostic indicators for non-union in osteoporotic bone

Indicator	Clinical presentation	Radiological features	Laboratory tests
Persistent pain	Ongoing localized pain at the fracture site	Lack of callus formation; visible fracture gap	Elevated bone turnover markers (e.g., alkaline phosphatase)
Abnormal mobility	Instability or abnormal movement at the fracture site	Sclerosis or hypertrophic bone at fracture ends	Low vitamin D levels, indicating poor bone metabolism
Absence of healing signs	Lack of progressive improvement in weight-bearing or function	Delayed or absent bridging of the fracture	Low bone density on DEXA scan

Management

Management of fracture non-union in osteoporotic bones requires a multifaceted approach addressing the impaired bone healing and the compromised bone quality characteristic of osteoporosis. The management typically involves optimizing bone biology, stabilizing the fracture, and enhancing the healing environment. Pharmacological interventions, such as bisphosphonates, teriparatides, or denosumab, are often employed to improve bone density and strength, directly targeting the underlying osteoporosis [15]. Surgical management remains the cornerstone for most cases of non-union, with options including revision fixation using locking plates or intramedullary nails and bone grafting to provide osteogenic, osteoinductive, and osteoconductive support [4,16]. Autologous bone grafts, allografts, and bone substitutes like demineralized bone matrix or calcium phosphate can be utilized depending on the clinical scenario. Adjunctive therapies, such as low-intensity pulsed ultrasound and extracorporeal shockwave therapy, have shown promise in stimulating bone healing in osteoporotic non-unions [17]. Addressing systemic factors like nutrition, vitamin D levels, and comorbidities is critical for optimizing outcomes. A tailored, patient-specific approach that integrates these strategies is essential for successfully managing fracture non-union in osteoporotic patients, minimizing complications, and enhancing functional recovery.

Innovative therapeutic approaches

Innovative therapeutic approaches to managing fracture non-union in osteoporotic bones are focused on enhancing bone regeneration, improving mechanical stability, and addressing the biological deficiencies inherent in osteoporotic bone. One emerging area is biologics, including growth factors like bone morphogenetic proteins (BMPs) and stem cell therapy. BMPs, such as BMP-2 and BMP-7, have shown potential in stimulating bone formation by enhancing osteoblastic activity and are increasingly being incorporated into surgical management strategies for non-union cases [5]. Stem cell therapies, including mesenchymal stem cells, are also gaining attention due to their ability to differentiate into osteoblasts and promote bone healing when delivered directly to the fracture site [7].

Another innovative approach is gene therapy, which introduces genes encoding growth factors to stimulate bone repair. Early studies suggest that delivering osteogenic genes locally can significantly enhance bone regeneration in non-union cases. In addition, advancements in biomaterial science have led to the development of bioactive scaffolds and synthetic bone graft substitutes that provide mechanical support and release therapeutic agents like growth factors to enhance healing [18]. Nanotechnology is also being explored, with nanoparticles engineered to deliver osteoinductive agents directly to the fracture site, promoting localized bone healing [19].

Combined with enhanced implants explicitly designed for osteoporotic bone and implant fixation techniques [20], these innovations offer new possibilities for managing challenging non-unions (Table 2). Although still evolving, these approaches promise to transform non-union management in osteoporotic patients, potentially reducing healing times and improving outcomes where traditional methods have been insufficient (Table 3).

**Table 2:** Comparison of traditional vs. innovative treatments for non-union BMP, bone morphogenetic protein

Category	Traditional treatments	Innovative treatments
Method of treatment	Open reduction and internal fixation	Growth factors (e.g., BMPs)
	Bone grafting	Stem cell therapy
Advantages	Widely available and well-established with consistent outcomes	Potential for faster healing through targeted biological enhancement
	Effective for structural support and immediate mechanical stability	Customized treatment options tailored to the patient’s biological needs
Challenges	Limited efficacy in osteoporotic bone due to compromised bone quality and poor integration	High cost, complex regulatory requirements, and limited long-term data
	Risk of hardware failure, infection, and delayed healing in osteoporotic patients	Technical difficulties and complexity in the application, storage, and procurement of cells

**Table 3:** Summary of emerging biologic and regenerative therapies BMP, bone morphogenetic protein; MSC, mesenchymal stem cell

Therapy	Mechanism of action	Current clinical evidence	Potential applications
BMPs	Stimulate osteoblast differentiation and activity, promoting bone formation	Shown effective in enhancing union rates in complex fractures [5]	It can be used for fractures with poor healing potential or high-risk patients
Stem cell therapy	MSCs differentiate into bone-forming cells at the fracture site	Early trials indicate improved bone healing and regeneration [20]	Particularly beneficial for non-unions in osteoporotic patients with poor biological healing response
Gene therapy	Introduces osteogenic genes to enhance bone growth and regeneration	Experimental studies show promise but limited human trials so far [8]	It could be applied in complex or refractory non-unions requiring biological enhancement

Future direction and clinical implications

The future direction in managing fracture non-union in osteoporotic bones centers on integrating advanced biologics, personalized medicine, and regenerative technologies. Emerging therapies like growth factors (e.g., BMPs), stem cell applications, and gene therapy are promising to enhance bone healing by targeting the underlying biological deficits in osteoporotic bones [5,6]. Additionally, innovations in biomaterials, such as bioactive scaffolds and nanotechnology, aim to improve mechanical stability and localized delivery of therapeutic agents [18,21]. 3D printing technology also creates customized implants that offer better fit and integration [22]. These developments pave the way for more personalized and effective treatment strategies, potentially reducing healing times and improving clinical outcomes. However, further research, clinical trials, and regulatory approval are essential to bring these therapies into widespread practice and ensure safety and accessibility. Overall, the evolving landscape of therapeutic innovations offers hope for more reliable and patient-specific management of fracture non-union in osteoporotic patients.

## Conclusions

Fracture non-union in osteoporotic bones remains a significant clinical challenge due to the complex interplay of compromised bone quality, impaired healing mechanisms, and the unique demands of treating an aging population. Traditional management approaches, while effective to some extent, are often insufficient in addressing the biological deficits in osteoporotic bone. However, emerging innovations in biologics, stem cell therapy, gene therapy, and advanced biomaterials pave the way for more targeted and effective interventions. Integrating these novel therapies with improved surgical techniques and personalized treatment strategies can transform care by promoting faster, more reliable healing while reducing complications. As these advances continue to evolve, they promise to significantly enhance outcomes for patients with osteoporotic fracture non-union. Nevertheless, successful translation into widespread clinical practice will require ongoing research, careful validation, and a multidisciplinary approach that considers each patient’s individual needs. By embracing these innovations, the future of treating osteoporotic fracture non-union is poised to be more effective, personalized, and patient-centered.
